# Assisted Reproductive Technology in Iran: The First National Report on Centers, 2011

**DOI:** 10.22074/ijfs.2016.5044

**Published:** 2016-09-05

**Authors:** Mehrandokht Abedini, Azadeh Ghaheri, Reza Omani Samani

**Affiliations:** 1 Deputy of Health, Department of Family Health, Ministry of Health and Medical Education, Tehran, Iran; 2 Department of Epidemiology and Reproductive Health, Reproductive Epidemiology Research Center, Royan Institute for Reproductive Biomedicine, ACECR, Tehran, Iran

**Keywords:** Iran, Assisted Reproductive Technology, Monitoring

## Abstract

**Background:**

Due to the worldwide increase in infertility, it is both necessary and important to have assisted reproductive technology (ART) registries. In Iran, donation and surrogacy programs are approved by decrees from religious scholars. ART has been used since 1984 in Iran and the first Iranian infant conceived by gamete intra-fallopian transfer (GIFT) was born in 1989. This report, however, is the first national report on Iranian ART centers.

**Materials and Methods:**

This cross-sectional study, conducted under the supervision of the Iranian Ministry of Health, presented a summary of the numbers and percentages of centers that provided infertility services in Iran, as well as the status of ART in Iran during 2011.

**Results:**

A total of 52 centers reported treatment cycles and performed approximately 29000 intrauterine insemination (IUI), in addition to 35000 *in vitro* fertilization (IVF) and intra-cytoplasmic sperm injection (ICSI) cycles.

**Conclusion:**

Iran has considerable potential to provide IVF services for both Iranians as well as other nationalities throughout the region. This proves the need for a national center that will implement a registry system.

## Introduction

With the expansion of infertility worldwide, the importance of assisted reproductive technology (ART) registries is critical. This registry information can assist health authorities, patients seeking medical assistance, the medical profession, and laboratory professionals in providing optimal patient care. A registry can provide the public a better understanding of ART procedures (1).

Reports of ART from European countries are presented annually with a four-year delay (2). The latest report of the European *in vitro* fertilization (IVF)-monitoring (EIM) has presented results of treatments initiated during 2010. Data is collected from existing national registries in the participating countries and directly entered by each national coordinator into the EIM database (3). ART data from the United States is also presented annually in surveillance papers (4). Data collected by the Latin American Registry of Assisted Reproduction (RLA) is also obtained from ART treatments performed in 155 institutions in 14 countries (5). Nevertheless, there is little information regarding the status of ART in Middle Eastern countries (6).

Iran is the only Islamic country in which donation and surrogacy programs are practiced. These programs have been accepted and approved by decrees from clergy scholars (7, 8). ART was first used in 1984 in Iran and the first Iranian infant conceived by gamete intra-fallopian transfer (GIFT) was born in 1989. Since then, more than 50 centers have been established in Iran. However, scant attention has been paid to a national registry and this is the first report on Iranian IVF monitoring.

This paper aims to present data gathered from the Iranian infertility centers in 2011. It proceeds by tracing the accessibility, procedure, cost, and some challenges of IVF in Iran. While definitions used in medically assisted reproduction are different in various settings, this paper has used the definitions suggested by the International Committee for Monitoring Assisted Reproductive Technology (ICMART).

## Materials and Methods

We gathered current registry information under the supervision of the Iranian Ministry of Health. This cross-sectional study dealt with the treatment outcomes during 2011 while the population of the country approximated 75.149 million. To collect data for this registry, we designed a form that used published literature and surveillance reports from American Society for Reproductive Medicine (ASRM), National Institutes of Health (NIH), and European Society of Human Reproduction and Embryology ESHRE (9, 10). The content of the questions was evaluated and approved by health and medical experts using the nominal group method and also by the Ethics Committee of the Iranian Ministry of Health.

In Iran, although infertility centers provide ART, clinics and hospitals also offer a portion of reproductive services. In Iran, all medical services provided in a hospital are under the supervision of a medical university. Thus, in order to obtain information from each center for completing the designed form, we sent there an expert familiar with basic concepts and definitions of ART from the medical university affiliated with the respective center. In this way, we contacted all centers and gathered data via the questionnaire.

Iran is divided into 31 provinces. This report provides data on the numbers of infertility centers, the numbers and types of provided services for ART, and the distribution of admitted clients according to the provinces of Iran in 2011. Specifically, this report provides summarized information of the numbers and percentages of centers that have provided infertility services based on the source of the egg (patient or donor) and the status of the embryos (fresh or thawed). Data also includes the number of treatments from standard IVF, intra-cytoplasmic sperm injection (ICSI), and intrauterine insemination (IUI) performed during 2011 in Iran.

Infertility clinics are divided according to their level of expertise: primary (level I), secondary (level II), and tertiary (level III). This report only focuses on level III infertility centers located throughout Iran. These centers have an ART laboratory which includes day care centers or centers in the hospitals that perform diagnostic and therapeutic functions at the highest level of specialization. In order to assess the level III criteria adjustment of these centers, data on the number of specialist staff or lack of specialists is provided in this report.

Hospitals and level III infertility clinics are integrated with medical universities in Iran. Iranian medical universities undergo an annual evaluation on the basis of research activities using criteria such as the numbers of faculty members and researchers, knowledge production and budget, and leadership and governance. Medical universities are then ranked according to three categories: type 1 with large numbers of academic staff and high levels of research funds, type 2 university with less staff and research funds compared to type 1, and type 3 with still fewer numbers of academic staff and lower research funds than type 2 universities (11). This report also presents the distribution of fertility centers based on the type of medical universities.

## Results

**Fig.1:**
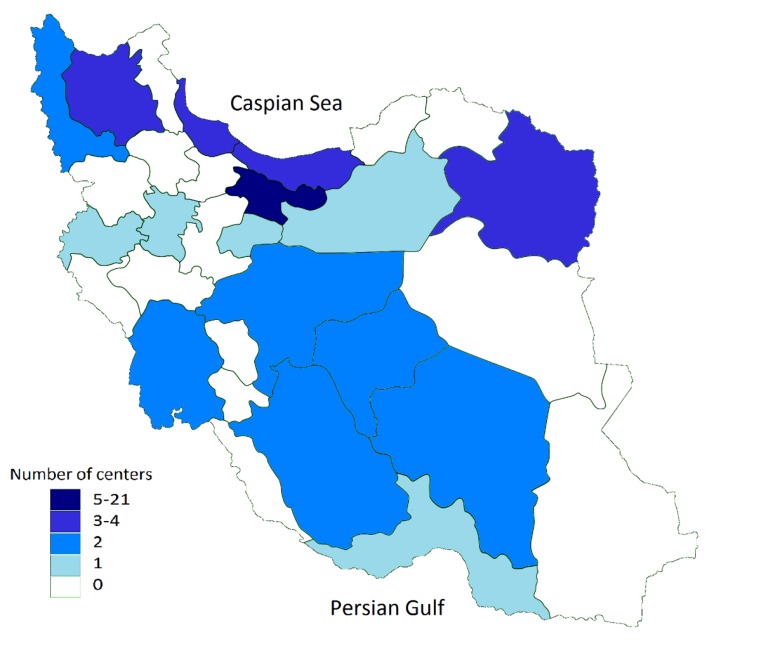
Distribution of infertility centers that performed assisted reproductive technology (ART) procedures in Iran during 2011.

In 2011 there were 52 infertility centers in Iran, with most located in major cities. The number of infertility centers that performed ART procedures varied by province and the type of university hospitals (Fig .1).

A total of 34 centers were affiliated with type 1 and 18 centers with type 2 university hospitals. Table 1 shows the distribution of infertility centers by the type of university hospital. Among all type 2 medical universities, Zahedan, Qazvin, Ardabil, and Gorgan did not have any level III infertility centers. From 46 medical universities in Iran, 18 had at least one level III infertility center (Table 1).

**Table 1:** List of medical universities and numbers of their affili- ated centers


Type 1 (n=34)	Type 2 (n=18)

Shahid Beheshti (16)	Gilan (3)
Tehran (5)	Mazandaran (2)
East Azerbaijan (4)	West Azerbaijan (2)
Mashhad (3)	Kerman (2)
Isfahan (2)	Yazd (2)
Shiraz (2)	Babol (2)
Khuzestan (2)	Hamadan (1)
	Kermanshah (1)
	Semnan (1)
	Qom (1)
	Bandar Abbas (1)


Medical university (No. of centers).

The infertility centers were divided into: private, government, and Academic Center of Education, Culture and Research [ACECR or the former Jihad Daneshgahi (non-government organizations)]. Among the 52 centers, there were 27 (52%) private, 21 (40%) government, and 4 (8%) ACECR.

**Fig.2:**
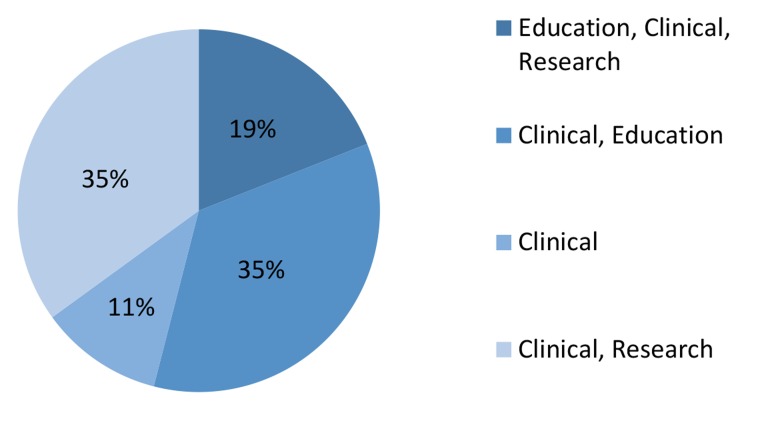
Percentage of clinics according to provision of services.

Medical universities of Iran provide personal, educational, and research services. Thus infertility centers can be categorized as having one or all of the following: educational, clinical, and research departments. The percentage of centers with respect to their services is presented in Figure 2.

**Fig.3:**
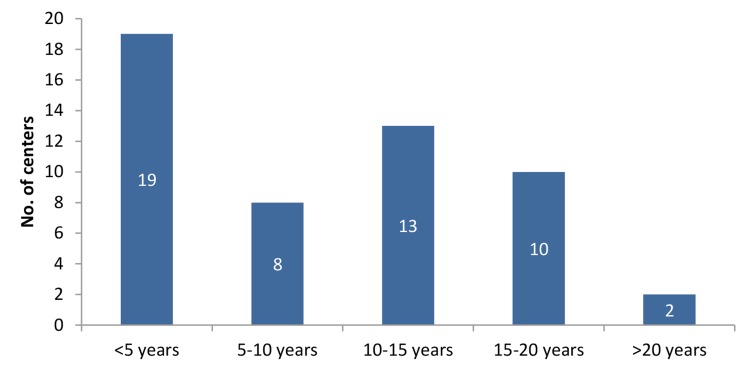
Frequency of clinics according to years of experience.

We have classified the centers according to the duration of their work. The oldest center is the government center of Yazd with 22 years of experience, followed by Shariati Hospital with 21 years, both of which are educational centers; after which are the private centers of Royan Institute with 20 years, Isfahan with 18 years, and Sarem and Navid centers, each with 17 years of experience (Fig .3).

**Fig.4:**
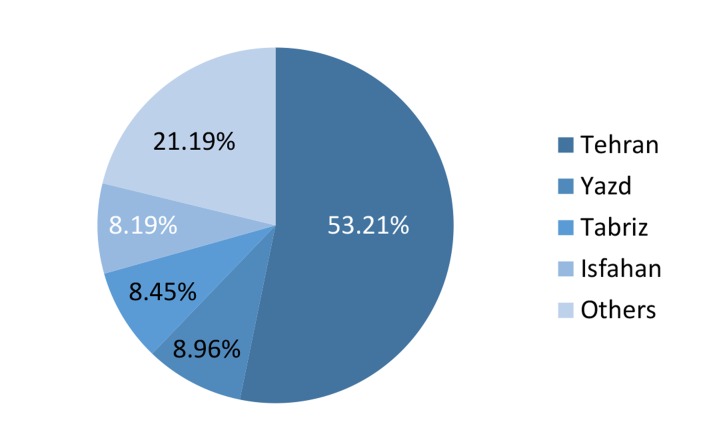
Percentage of visited clients in infertility centers of Iran during 2011.

There were approximately 29000 IUI, as well as 35000 IVF and ICSI cycles in Iran during 2011. The sum of average number of visits in tertiary infertil- ity centers for any type of diagnostic and therapeu- tic process per month was 39063; the mean num- ber of new admissions among the centers was 641 per month. The minimum number of admissions in centers was three per month. The maximum num- bers of admissions occurred in three private centers in Tehran and Isfahan. A total of 21 centers were located in Tehran, the capital of Iran, with a total number of 20786 clients per month, which was the highest proportion (53%) in the country (Fig.4). 

Table 2 shows the number of centers according to provided services. All centers had documented records of patients’ general information; however, 17 (33%) had electronic records. The monthly rate of ART ser- vices provided by centers is shown in Table 3.

**Table 2:** Numbers of centers that provide different ART services


Type of ART service	Number of centers (%)

IUI	52 (100 )
IVF	49 (94)
ICSI	50 (96)
Surrogacy	35 (67)
Egg donation	43 (83)
Embryo donation	35 (67)
Sperm donation	1 (2)
Sperm bank	6 (12)
Embryo freezing	48 (92)
Egg freezing	35 (67%)
Sperm freezing	40 (77%)
Diagnostic laparoscopy and hysteroscopy	44 (85%)
Therapeutic laparoscopy and hysteroscopy	42 (81%)
Male infertility surgeries	43 (83%)


ART; Assisted reproductive technology, IUI; Intrauterine in- semination, IVF; In vitro fertilization, and ICSI; Intra-cytoplasmic sperm injection.

In 2011, the mean cost of IVF ranged from $2250 to $3600 in government and private centers.

In Iran, technical managers of all centers should be fellowship of infertility or qualified by having three years of experience in an infertility center. Therefore, having infertility fellowship was assessed in all cent- ers as criteria. In 5 centers, an infertility fellowship did not exist, 3 had no urologists, and 2 did not have any embryologists. In numerous cases, one embryologist simultaneously cooperated with more than one center. In 16 centers, midwives were used as alternatives to nurses and 2 centers had no midwives. 

**Table 3:** Monthly rate of assisted reproductive technology procedures in 2011


Type of service	Mean	Minimum	Maximum

Intrauterine insemination (IUI)	47	2	300
In vitro fertilization (IVF)	57	0	400
	Total in all centers*	Minimum*	Maximum*
Surrogacy	165	1	61
Egg donation	997	1	150
Embryo donation	545	2	300


*; Mar. 21^st^ to Sept. 22^nd^, 2011.

A total of 36% of centers had neither an internal specialist nor an endocrinologist. Patients who needed specialized medical counseling (i.e., urology, endocrinology, and genetics) were referred to another clinic or hospital when there was no specialist located at that center. Some infertility centers presented their needs for specialists (Table 4).

**Table 4:** Number of centers which required different medical specialists


Type of required specialist	Number of centers (%)

Gynecologists with infertility focus/infertility fellowship	8 (15)
Urologist	3 (6)
Embryologist	11 (21)
Internal specialist or an endocrinologist	19 (36)
Psychologist	32 (62)
Genetics specialist	30 (58)
Nutrition specialist	25 (48)


In order to assess level III criteria adjustment for diagnostic tests, we checked the sonohysterography and hysterosalpingography performances. In 31 centers, sonohysterography and hysterosalpingography were performed. All centers with the exception of one center, were equipped with a microinjection device.

Among 28 infertility research centers, 7 had scientific research journals: Vali Asr, Royan Institute, Avicenna Infertility Clinic, Family Hospital, Mehr Infertility Center, Sarem Hospital, and Mother Infertility Center.

## Discussion

The increased use of reproductive science developments, as well as the least legal or religious barriers to ART in Iran as an Islamic country, have raised the hopes of infertile couples.

The availability of infertility services, as a product of public and private health policies, could determine the allocation of personnel, equipment, and facilities (12). According to our data, the number of infertility centers that performed ART varied considerably by province. Residents of Tehran with 21 infertility centers had the easiest access to infertility centers followed by East Azerbaijan, Khorasan Razavi, Gilan, and Mazandaran with 4 or 3 centers. The remainder of provinces had none to 2 centers. Lack of geographic access to level III infertility services has obviated the necessity for increasing the availability and utilization of these services in different areas of the country. Establishing at least one level III center in all provinces rather than multiple centers in specific areas could provide extensive facilities to cover the entire country.

Most medical universities that had an infertility center were type I. All centers provided clinical services, however 35% also had educational and research departments. Numerous centers solely focused on clinical activities (35%), which have suggested that stronger links among research, education, and practice are needed.

Although 12 centers had more than 15 years of experience, most had less than 5 years of experience in providing ART services. Compared with the most experienced infertility clinics in the US, which were established in the early 1980s, our first infertility center had almost a decade delay in establishment.

The estimated number of IVF per million population in 2011 showed that the national utilization of IVF was less than the equivalent reported from European countries in 2010 and the US in 2011 (10, 13). In 2011, most centers offered IUI, IVF, and ICSI. The number of centers that offered egg and embryo donation was almost half compared with clinics in the US. Only one center reported that they offered sperm donation to clients, which was entirely different from similar percentage of this service in the US (14).

It seems that due to lack of a supporting law in the country, some centers did not claim their donation practice. Due to legal and legislative approval of embryo donation in Iran, as well as the absence of laws regarding sperm donation, our data about embryo donation might be more realistic (8, 15). This could cause centers to perform embryo donation instead of sperm donation and as a result the portion of embryo donation cycles might increase compared with other donation programs. The low percentage of centers that provided sperm banks could be another consequence of a vague law and legislations on this program.

The mean number of new monthly admissions among centers was 641. As far as we know this has not been reported in other countries’ studies. Usually the number of cycles is reported for each infertility clinic. Lack of a referral system in Iran may lead to overestimation of the number of new admissions because a person as a new admission of a specific infer- tility center may refer to another center after a period of time and be counted in more than one center.

Worldwide, ethical issues in reproductive medi- cine and assisted reproduction are influenced by religions. In Iran, Islamic primary sharia. In Latin America, the Catholic Church applies considerable pressure to prevent access to IVF and third party assisted reproduction is banned (8). Studies have mostly focused on the view of Sunni Islam on ART, as they comprise 90% of the Muslim population. Shia Islam which is concentrated in Iran, Azerbaijan, Iraq, and Afghanistan relies on clergy scholars’ decrees on different new issues including ART. Although there are differences in the decrees, all are correct for the scholars’ followers. Some Shia scholars have approved ART using third party and the embryo donation law was passed by the Iranian parliament in 2003. In contrast, in 1986, the Islamic Fiqh Academy based in Jeddah (Majmaal-Fiqh al-Islami) considered all types of third party assisted reproduction forbidden (haram) (8).

Ethical conditions may lead to legislative rules that are adopted by legal experts, ethicists, religious scholars, and politicians. Therefore, some people may obtain their desired infertility treatment outside of their own countries. In this way, they travel from a restrictive country to a permissive one (16, 17). Infertility is highly stigmatized in developing countries and leads to profound social consequences for these couples. Hence, infertility is sometimes kept secret. Due to losing both social support and social capital, patients seek reproductive services in neighboring countries (18, 19). Since Iran is a Muslim country in which gamete and embryo donation are practiced as well as surrogacy, it can be the first choice for Muslims from other countries who seek infertility treatment. We have not gathered international patients’ data, so no conclusion could be made in this regard.

In most countries, cost is a serious concern for couples who receive infertility services. The cost of ART treatment is different worldwide due to the costliness of underlying health care systems and the level of patients’ subsidization. As all infertility centers in Iran operate outside of governmentfinanced health facilities, they actually provide services only for patients that can pay out of pocket for ART treatments. Although ART is cost prohibitive in Iran, the cost is relatively lower than neighboring countries with better economic situations and stronger currencies. The relative cost differences encourage infertile couples to travel to Iran to undergo ART (20, 21).

Considering the rate of lifetime experience of infertility (6.4%), as well as the rate of primary (21.1%), and secondary infertility (7.8%) in Iran (22), it is essential to draw up a national guideline. This guideline can offer the most practical advice on assisting couples with infertility problems, and take into account individual needs and preferences (14). Lack of national auditing, supervision, and a registry are the major drawbacks of this system. A national center is required to implement a registry system that reports important national outcomes of infertility centers such as success rates, numbers of embryos transferred, numbers of frozen-thawed eggs, and the woman’s age at the time of retrieval, in addition to an introduction to the infertility centers and costs for ART cycles. A national registry and monitoring can lead to improvement in quality of aspects of the structures, processes, and outcomes of infertility centers. Establishing this registry system can be initially implemented by developing audit activity and outcome committees in the centers.

## Conclusion

This paper has presented the status of ART in Iran during 2011. The most obvious finding to emerge from this study is that Iran has great potential to provide IVF services for both Iranians and other nationalities throughout the region. Therefore the implementation of a registry system seems to be vital.
